# Systematic whole-genome sequencing reveals an unexpected diversity among actinomycetoma pathogens and provides insights into their antibacterial susceptibilities

**DOI:** 10.1371/journal.pntd.0010128

**Published:** 2022-07-25

**Authors:** Andrew Keith Watson, Bernhard Kepplinger, Sahar Mubarak Bakhiet, Najwa Adam Mhmoud, Jonathan Chapman, Nick EE Allenby, Katarzyna Mickiewicz, Michael Goodfellow, Ahmed Hassan Fahal, Jeff Errington

**Affiliations:** 1 Centre for Bacterial Cell Biology, Biosciences Institute, Newcastle University, Newcastle upon Tyne, United Kingdom; 2 The Mycetoma Research Centre, University of Khartoum, Khartoum, Sudan; 3 Odyssey Therapeutics Inc, The Biosphere, Draymans Way, Newcastle Helix, Newcastle upon Tyne, United Kingdom; 4 School of Natural and Environmental Sciences, Newcastle University, Newcastle upon Tyne, United Kingdom; University Hospitals Sussex NHS Foundation Trust, UNITED KINGDOM

## Abstract

Mycetoma is a neglected tropical chronic granulomatous inflammatory disease of the skin and subcutaneous tissues. More than 70 species with a broad taxonomic diversity have been implicated as agents of mycetoma. Understanding the full range of causative organisms and their antibiotic sensitivity profiles are essential for the appropriate treatment of infections. The present study focuses on the analysis of full genome sequences and antibiotic inhibitory concentration profiles of actinomycetoma strains from patients seen at the Mycetoma Research Centre in Sudan with a view to developing rapid diagnostic tests. Seventeen pathogenic isolates obtained by surgical biopsies were sequenced using MinION and Illumina methods, and their antibiotic inhibitory concentration profiles determined. The results highlight an unexpected diversity of actinomycetoma causing pathogens, including three *Streptomyces* isolates assigned to species not previously associated with human actinomycetoma and one new *Streptomyces* species. Thus, current approaches for clinical and histopathological classification of mycetoma may need to be updated. The standard treatment for actinomycetoma is a combination of sulfamethoxazole/trimethoprim and amoxicillin/clavulanic acid. Most tested isolates had a high IC (inhibitory concentration) to sulfamethoxazole/trimethoprim or to amoxicillin alone. However, the addition of the β-lactamase inhibitor clavulanic acid to amoxicillin increased susceptibility, particularly for *Streptomyces somaliensis* and *Streptomyces sudanensis*. *Actinomadura madurae* isolates appear to have a particularly high IC under laboratory conditions, suggesting that alternative agents, such as amikacin, could be considered for more effective treatment. The results obtained will inform future diagnostic methods for the identification of actinomycetoma and treatment.

## Author summary

Mycetoma is a common health and medical problem that is endemic in many tropical and subtropical countries and has devastating effects on patients. The destructive nature of late-stage infection means that treatment often requires long term use of antibiotic therapy, massive surgical excisions and amputation. Several different bacterial species have been described as causing this disease but our understanding of the true diversity of mycetoma causing bacteria has been limited by a lack of molecular sequence data. We have now sequenced the genomes of 17 samples isolated from patients at the Mycetoma Research centre in Sudan, revealing a diverse range of species associated with infection including one new *Streptomyces* species, and three species with no previous association with human mycetoma. Crucially, all isolates had a high IC against the current first-line antibiotics used to treat actinomycetoma under laboratory conditions. The highest ICs were seen in *Actinomadura madurae*, which was also the most frequently observed species isolated from patients in our study. We hope that these results will aid in the development of future rapid diagnostic tools and the improvement of treatment outcomes.

## Introduction

Mycetoma, a neglected tropical disease, is a chronic subcutaneous granulomatous inflammatory disease [1,2]. The inflammatory process usually spreads to affect the skin, deep tissues and bone, leading to massive destruction, deformity and disability, and can be fatal [3–5]. It is endemic in many countries around the equator in a zone often described as the mycetoma belt, with Sudan reported as the most affected country [6–8].

Mycetoma is characterised by a painless mass with multiple sinuses which discharge material including grains which are colonies of the causal agents. Colours, sizes and consistency of the grains can often be indicative of the aetiological agent [9,10]. It can affect different body parts but is most commonly seen in the feet and hands. Young adults and children are frequently affected, leading to numerous negative medical, health and socioeconomic impacts on patients and their families [5,11]. The combination of the painless nature of the initial stages of the disease, low availability of health facilities in endemic regions and the low socioeconomic status of those infected explains the late presentation of patients at clinics. Consequently, extensive surgical intervention often becomes the only remaining option for treatment [12–14].

While mycetoma infections are characterised by a shared phenotype, a broad taxonomic range of species have been described as causative agents, including fungi producing eumycetoma and actinobacteria causing actinomycetoma [6,15,16]. Actinobacteria are filamentous bacteria that are universally distributed and are found in terrestrial and aquatic settings, but primarily associated with soil environments where they play an important role in the decomposition of organic material. They are also an important source of specialized metabolites such as antibiotics. A relatively small number of actinobacterial species are known to cause actinomycetoma. The most common causal agents of actinomycetoma are *Actinomadura madurae*, *Actinomadura pelletieri*, *Nocardia asteroides*, *Nocardia brasiliensis* and *Streptomyces somaliensis* [17]; less well known species include *Actinomadura latina* [18,19] *Actinomadura mexican*a [20], *Streptomyces albus* [21], *Streptomyces griseus* [22] and *Streptomyces sudanensis* [23]. Whilst *S*. *sudanensis* and *S*. *somaliensis* are closely related, other mycetoma agents within the *Streptomyces* genus are part of distinct taxonomic lineages, highlighting the diversity of mycetoma agents within this genus [24].

The genera *Actinomadura* and *Streptomyces* are typically associated with growth in soil rather than with pathogenesis. Indeed, the primary route of transmission for actinomycetoma agents is thought to be the traumatic inoculation of bacteria into the subcutaneous tissue from soil particles (e.g. bare feet punctured by thorns or splinters). There is also evidence that *Streptomyces* can cause mycetoma in animals [25,26], as well as fistulous withers in donkeys [27]. Historically, mycetoma diagnosis has been based mainly on clinical presentation, surgical biopsies and histopathological examination of grains in the mycetoma granuloma mass and on colony characteristics on culture media, but only in a few centres [28–30]. Unfortunately, these features often overlap between different causative organisms, making diagnosis challenging [31,32].

Accurate diagnosis and the ability to distinguish between actinomycetoma and eumycetoma is vital, as the treatments for them are fundamentally different [28–30]. Current treatment for eumycetoma involves long term anti-fungal medication and surgical intervention. On the other hand, actinomycetoma is treated with a combination of antibiotics. However, prolonged treatment is required to effect a cure, and the response to treatment is variable depending on the causative agent and associated drug resistance [33–36].

It is important to identify the causative agents of actinomycetoma to ensure they respond to medical treatment. Since actinomycetoma is caused by several species, it seems likely that molecular differentiation within and between species may reveal differences in responses to frontline treatments and that adaptation of treatments would be beneficial for patient care. We, therefore, set out to obtain whole-genome sequences of a variety of actinomycetoma pathogens, determined their taxonomic status, and characterised their IC profiles to commonly used antibiotics.

## Materials and methods

### Ethics statement

Ethics approval for this study was obtained from the Mycetoma Research Centre, Khartoum, Sudan IRB (Approval no. SUH 11/12/2018). Written informed consent was obtained from each adult patient and parents or guardians of the population under 18 years old. Confirmed mycetoma cases were referred for management at the Mycetoma Research Centre (MRC).

### Sample isolation and subculture

The patients were seen at the Mycetoma Research Centre (MRC), University of Khartoum. Granulomatous material from mycetoma lesions were obtained by surgical biopsy. The grains were washed three times in sterile normal saline, plated onto yeast extract agar (YEA) and incubated at 37°C for one to two weeks under aerobic conditions. Once microbial growth was visible, the colonial morphology was recorded, and Gram-stained smears prepared from each isolate. Actinomycetoma organisms, unlike their eumycetoma counterparts, are characterised by their typical filamentous Gram-positive appearance.

Strains (17 in total) isolated from separate patients, received at Newcastle University from the MRC were cultivated on tryptic soy agar and oatmeal agar (20 g/l of oat was boiled in water for 20 min; the liquid was strained using a sieve, and 20 g/l of agar was added prior to autoclaving) at 30°C.

### Histopathology and cytology

Deep excisional biopsies were taken from the mycetoma lesions and preserved in 10% neutral buffer formalin. The tissue biopsies were processed and then cut using a rotary microtome (Leica, Germany). The 3–5μm sections obtained were stained with haematoxylin and eosin (H&E), and the slides examined using a light microscope (Olympus, Germany) for the presence of grains and the type of inflammatory reactions according to previously described criteria [30].

Cytological smears were prepared on aspirated materials from mycetoma lesions using a 23 gauge needle attached to a 10 ml syringe. The smears were allowed to air dry and then stained with May-Grünwald-Giemsa (MGG) and H&E stains. The slides were then examined by light microscopy for the presence of grains of the causal agents using previously described criteria [37].

### DNA isolation, sequencing and assembly

DNA was isolated from strains using a modified version of the "salting-out" method [38]. A 15 mL culture grown in TSB was harvested by centrifugation and resuspended in 5ml of SET buffer, containing a final concentration of 1.5 mg/ml lysozyme, and incubated at 37°C for 1.5 h. RNase was added to a final concentration of 20 μg/ml and the sample incubated at room temperature for 1 min. Pronase (final concentration 0.5 mg/ml) and SDS (final concentration 1%) were added and mixed by inversion at 37°C for 2 h. 2 ml of 5M NaCl and 5 mL chloroform were added, with incubation at room temperature for 30 min. Phases were separated by centrifugation, and the aqueous phase transferred into a fresh tube. The DNA was precipitated by the addition of 0.6 vol of propan-2-ol. The high molecular weight DNA was spooled around a sealed Pasteur pipette. The DNA was washed using freshly prepared 70% ethanol before being allowed to air dry. The DNA was finally dissolved in 10 mM Tris-HCl pH 8.5.

For all isolates acquired from the Mycetoma Research Centre, the libraries for Minion sequencing were prepared using a ligation sequencing kit with the native barcode extension kit, according to the protocols of the manufacturer. Sequencing was performed on two Flo-MinSP6 flow cells. Libraries for Illumina sequencing were prepared using the Illumina DNA prep kit with Nextera DNA CD Indexes. Libraries were sequenced on an ISeq 100. Nanopore reads were base-called and demultiplexed using guppy 3.4.4+a296acb. Draft genome assemblies from nanopore sequences were generated using flye 2.8.2 [39], followed by consensus correction using Medaka 1.2.1 [40] implemented in the "demovo" pipeline (kindly provided by Demuris Ltd). Illumina reads were mapped to the draft Nanopore assembly using minimap 2.17 [41], and pilon 1.23 was used to correct the genome assembly based on the Illumina mapping. This process was repeated four times. Assemblies of all isolate genomes are deposited on NCBI under BioProject ID PRJNA782605. Diamond [42] and MEGAN [43] were used to correct frameshifts by inserting N characters to the genome assemblies as described previously [44]. These assemblies are available via FigShare (https://doi.org/10.6084/m9.figshare.19447358). Finally, genome assemblies were annotated using the NCBI pgap pipeline [45].

### Type-strain genome sequencing and assembly

Type strains of *S*. *somaliensis* (DSM 40738) and *S*. *sudanensis* (DSM 41923) were acquired from DSMZ. Strains were cultured and DNA isolated as previously described for isolate genomes. Illumina sequencing was carried out by the NU-OMICS facility at Northumbria University, while ONT sequencing was carried out by Demuris Ltd as previously described for isolate genomes. The genomes were assembled using Canu [46] and corrected with Illumina data using bowtie2 [47]. These assemblies are deposited on NCBI under BioProject ID PRJNA824685.

### 16S rRNA taxonomy

The longest 16S rRNA annotated in each isolate genome was used as a query in the EzBioCloud 16S rRNA database. Isolates were presumptively assigned to the genera *Actinomadura* or S*treptomyces* based on their best hits in this database as determined by sequence identity. The 50 most similar rRNA sequences to each isolate sequence were extracted from the database, pooled according to the predicted genus of the corresponding isolates, and dereplicated. This resulted in the generation of 16S rRNA with similarity to *Actinomadura* and *Streptomyces* related isolates. To explore whether the causative agents of actinomycetoma are found in the environment in Sudan, as might be expected from the described route of transmission, all *Streptomyces* 16S rRNA sequences from a recent survey of soil samples in Sudan were examined [48]. Additionally, 11 samples (NCBI accessions: EU544231.1 to EU544241.1), isolated from humans and animals in Sudan, were included in the *Streptomyces* dataset [48].

The 16S rRNA sequences were aligned using mafft7.453 (—auto mode) [49], and the alignment trimmed using trimal 1.4 (—automated1) [50]. Phylogenetic trees were inferred with iqtree2 using the best scoring GTR model designated by iqtree’s automated model selection [51]. All phylogenetic trees were midpoint rooted and visualised using iToL [52].

### Whole genome taxonomy

GTDB-Tk [53] was used to classify genomes based on whole-genome data using two methods: maximum-likelihood placement of genomes into a reference phylogeny based on the bac120 set of 120 marker genes using pplacer [54] and by comparing the average nucleotide identity (ANI) of isolate genomes to those in GTDB [55] using FastANI [56].

The genomes of all “close relatives” of isolates identified by GTDB-tk were extracted from the database. Further, the protein sequences of genes from the GTDB bac120 set of phylogenetic markers [55] were extracted from all isolates, “close relative” GTDB genomes, and type strain genomes. As with 16S rRNA, the dataset was separated into *Actinomadura* and *Streptomyces* related isolates. All markers identified as a single copy in isolate genomes were retained for phylogenetic analysis. These marker sequences were individually aligned using mafft (—auto mode) and trimmed using BMGE (BLOSUM30 model) [57]. Phylogenetic trees based on the concatenation of the trimmed alignments were inferred using iqtree2 [51] with the C20 model [58]. All phylogenetic trees were midpoint rooted and visualised using iToL [52].

FastANI 1.32 [56] was used to estimate the Average Nucleotide Identity (ANI) of all pairs of isolated genomes, reference genomes from type strains of known causes of human actinomycetoma, and the genomes of close relatives to the isolates identified by GTDB-tk, thereby providing a measure of their diversity at the whole genome level. Average Amino acid Identity (AAI) values were calculated between isolates and any close relatives from GTDB with an ANI >90% using EzAAI [59]. Finally, DDH values between isolates and the single genome from GTDB identified by all previous methods as their closest relatives were estimated using GGDC [60]. ANI >95% [61], AAI >95% [62] and DDH > 70% [63] are the typical values used to delineate species boundaries.

### Genome size comparisons

Metadata from the Genome Taxonomy Database (version R06-RS202) was used to investigate the overall distribution of genome sizes within the *Streptomyces* genus (with taxonomy defined by the GTDB taxonomic classification [55,64]). A quality score was assigned to each genome (checkM completeness score—(5*checkM contamination score)) as previously used to assess genome quality [65,66]. The best quality genome for each species within the genus *Streptomyces* with a minimum quality score of 90% was included in the dataset. In total, 662 genomes from across the *Streptomyces* genus met these criteria.

### Antimicrobial susceptibility assay

The bacterial strains were plated onto Mueller-Hinton agar (Oxoid) except for MRC003, MRC013 and MRC019, which grew poorly on this medium and so were plated onto TSA (Difco). MRC008 did not grow as a lawn under any of the conditions tested and was excluded from the analyses. Antimicrobial susceptibility disks (Oxoid) were loaded with the following compound concentrations: amikacin (30 μg), amoxicillin (25 μg), amoxicillin/clavulanic acid (30 μg), erythromycin (15 μg), gentamicin (30 μg), rifampicin (5 μg) and trimethoprim/sulfamethoxazole 1:19 (25 μg). The disks were placed on the plates immediately after inoculation and incubated at 37°C for five days before measuring zones of inhibition in mm. Sequence-based predictions of antimicrobial resistance profiles were made using AMRfinderPlus [67].

## Results

### Isolation of actinomycetoma pathogens

The grains for culture were collected from confirmed mycetoma patients seen at the Mycetoma Research Centre, University of Khartoum. The patients were from Sudan, except for a patient from Yemen. All patients had meticulous clinical interviews and examinations after giving written consent. In addition, all of them had surgical biopsies taken under local or general anaesthesia. Microbial cultures obtained from grains were Gram-stained to distinguish actinobacterial from fungal pathogens. Seventeen independently isolated strains identified as filamentous actinobacteria were sent to Newcastle University for further analysis by whole-genome sequencing. All whole genome sequences are deposited on NCBI under BioProject ID PRJNA782605.

### 16S rRNA analyses reveal a high diversity amongst clinical isolates

**Fig 1:**
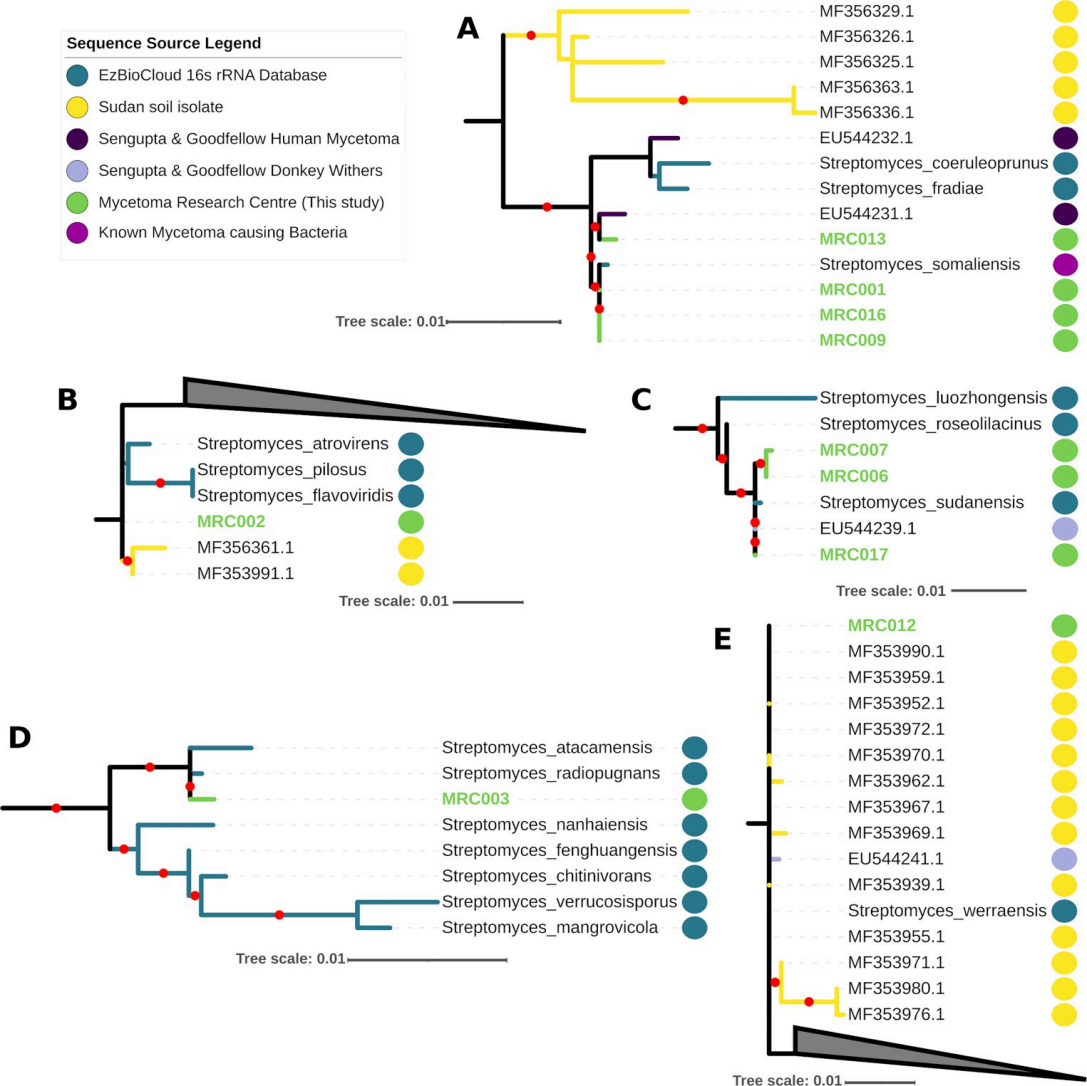
Extracts from the 16S rRNA phylogenies of *Streptomyces* (full figure in S1 Fig and https://itol.embl.de/shared/1MX60mtB0Ohk3) inferred using iqtree2 and the GTR+F+R5 model showing the placement of isolates (green dots) with related rRNA sequences from the ezBioCloud 16S rRNA database (blue), soil isolates from Sudan (yellow dots [48]) and isolates collected by Sengupta, Goodfellow and Hamid (NCBI accessions EU544231.1 to EU544241.1 from human actinomycetoma (purple) and from donkey withers (lilac). A red dot on branches indicates ultrafast bootstrap support >95. Triangles are used to represent collapsed clades.

The initial taxonomic assignment of isolates based on 16S rRNA sequence similarity supported their annotation as actinomycetes, with 10 isolates assigned to the genus *Streptomyces* and seven isolates assigned to the genus *Actinomadura*. Of the *Streptomyces* isolates, the 16S rRNAs of 7 of them were most similar to known causes of actinomycetoma; *S*. *sudanensis* (3 isolates; MRC006, MRC007 and MRC017) and *S*. *somaliensis* (4 isolates; MRC001, MRC009, MRC013, MRC016). These sequences also formed strongly supported clades with reference rRNA sequences in the phylogenetic analyses (Fig 1A and 1C). The initial taxonomic assignment of three isolates from the genus *Streptomyces*, MRC002, MRC003 and MRC012, did not correspond to previously identified sources of human actinomycetoma, with each branching separately in the 16S rRNA tree (Fig 1B, 1D and 1E), suggesting they may represent three independent new sources of human actinomycetoma from within the genus *Streptomyces*. These relationships are discussed in more detail in light of the whole genome data considered below. Isolates related to *S*. *somaliensis* (Fig 1A), as well as potentially novel actinomycetoma agents MRC002 and MRC012, were part of clades with 16S rRNA identified in Sudan soil samples [48], consistent with the described primary route of infection by traumatic inoculation of bacteria from the environment. Interestingly, the clade including the potentially novel MRC012, and the clade including *S*. *sudanensis* isolates, both also included 16S rRNA sequences from strains isolated by Sengupta, Goodfellow and Hamid from lesions of donkeys with fistulous withers in Sudan (Fig 1C and 1E; NCBI accessions: EU544241.1, EU544239.1), suggesting that these strains may be capable of infecting multiple hosts thereby raising the possibility of cross-species transmission of actinomycetoma.

**Fig 2:**
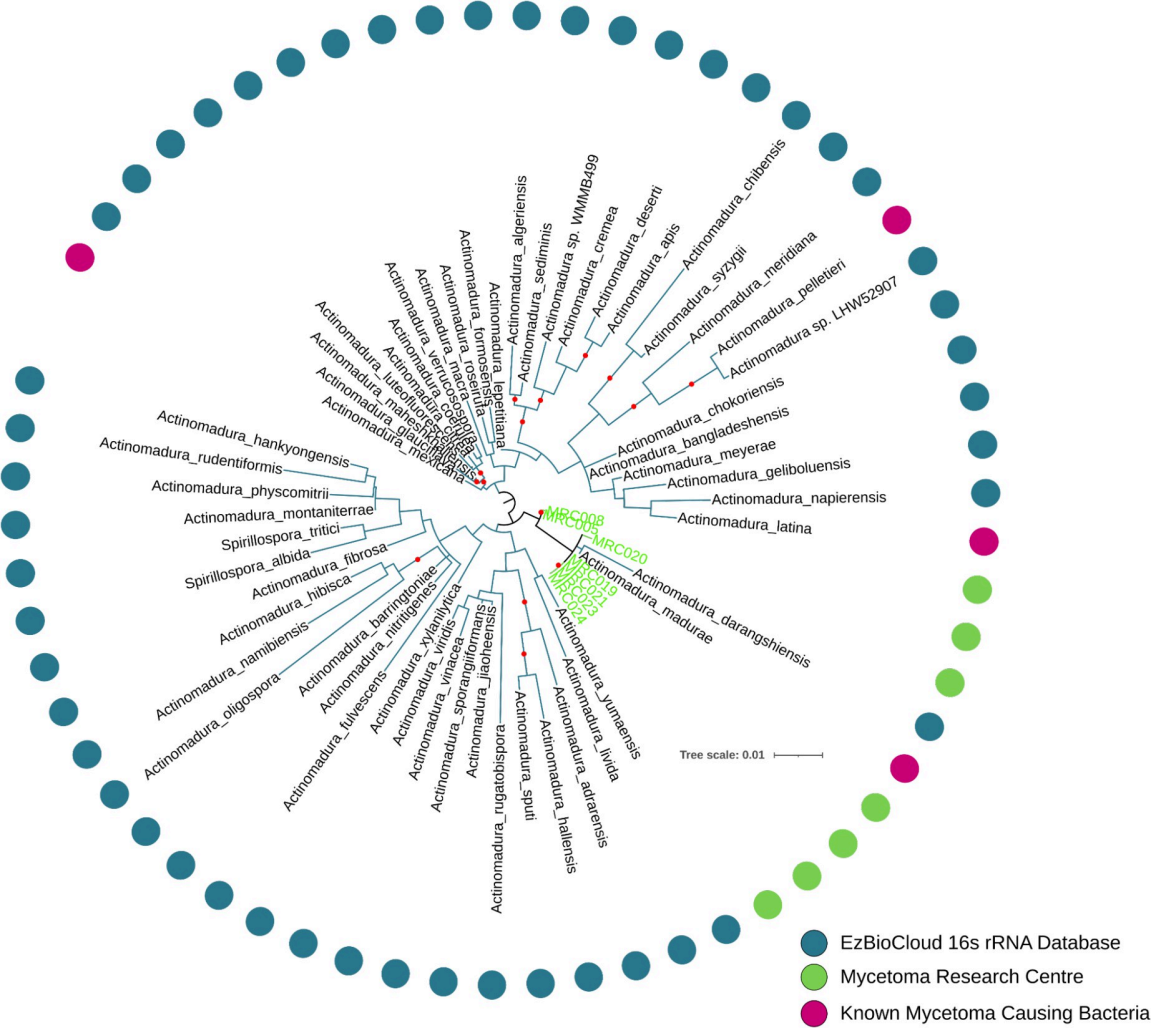
The 16S rRNA phylogeny of *Actinomadura* showing the placement of isolates (green) with related rRNA sequences from the ezBioCloud database (blue; pink for known pathogens). Inferred using iqtree2 and the GTR+F+R4 model. A black dot on branches indicates ultrafast bootstrap support >95. All isolates form a clade with *Actinomadura madurae* and *Actinomadura darangshiensis*, though support for this grouping is low.

Six of the seven isolates assigned to the genus *Actinomadura* were from regions across Sudan; the remaining one was from Yemen (MRC019). The 16S rRNAs of six of the isolates were most similar to the *A*. *mexicana* 16S rRNA sequence in the EzBioCloud database (>99% identity); the remaining one was closest to the reference *A*. *madurae* 16S rRNA. In the 16S rRNA phylogeny, all 7 isolates assigned to *Actinomadura* formed a weakly supported clade with *A*. *madura*e (a well-known cause of human mycetoma) and *Actinomadrua darangshiensis* (Fig 2), with MRC005 and MRC008 branching at the base of the clade. However, the weak support for this clade, means that relationships between the isolates and *A*. *mexicana* cannot be excluded. The precise nature of the relationships between these isolates, as well as all other isolates in our dataset, and existing strains were disentangled using corresponding whole genome sequence data.

### Whole genome analyses support the classification of *S*. *somaliensis* and *S*. *sudanensis* as separate species

In 2008, *S*. *sudanensis* was proposed as a new species closely related to *S*. *somaliensis* based on a combination of 16S rRNA sequence, DNA:DNA relatedness and phenotypic data [23]. Within the Genome Taxonomy DataBase (GTDB), two genomes are annotated as deriving from *S*. *somaliensis and S*. *somaliensis_A*, annotated as two separate species despite initially being described as deriving from the same *S*. *somaliensis* type strain in NCBI (DSM 40738^T^). These are GCF_012396115.1 and GCF_000258595.1. GCF_000258595.1 corresponds to the initially reported draft genome of *S*. *somaliensis* [68]. GCF_012396115.1 was more recently sequenced and deposited by the Bacterial Pathogens Special Branch (CDC), and is marked as having an “unverified source organism” by NCBI. In order to untangle the relationships between these genomes, we acquired the type strains of *S*. *somaliensis* and *S*. *sudanensis* from DSMZ (DSM 40738 and DSM 41923) and sequenced and assembled their genomes. Based on a combination of ANI, AAI and DDH values, the reported draft genome of *S*. *somaliensis* GCF_000258595.1 [68] is highly similar to our *S*. *sudanensis* type strain genome, which is consistent with its assignment to that species rather than to *S*. *somaliensis* [68] (Table 1). Furthermore, comparison of these genomes provided additional evidence that *S*. *somaliensis* and *S*. *sudanensis* should be considered as separate species [23], as ANI, AAI and DDH values are below the thresholds typically used to assign closely related strains to the same species (Table 1).

**Table 1:** Summarising the pairwise ANI, AAI and estimated DDH values of the *S*. *somaliensis and S*. *sudanensis* type strain genomes compared to genomes in GTDB. Species thresholds are usually defined by ANI >95%, AAI >95% and DDH >70%. Values that fall below these thresholds are highlighted in grey. Results indicate that GTDB GCF_000258695 (annotated as *S*. *somaliensis*) is most closely related to *S*. *sudanensis*, though it is a different species.

Genome 1	Genome 2	ANI (%)	AAI (%)	DDH (GGDH)
*GTDB S*. *somaliensis* (GCF_012396115)	*S*. *somaliensis* Type Strain Genome	99.6	99.7	98.4
*GTDB S*. *somaliensis* (GCF_012396115)	*S*. *sudanensis* Type Strain Genome	92.0	90.2	43.3
*GTDB S*. *somaliensis* (GCF_000258595)	*S*. *somaliensis* Type Strain Genome	91.9	89.9	42.2
*GTDB S*. *somaliensis* (GCF_000258595)	*S*. *sudanensis* Type Strain Genome	99.8	99.9	98.5
*GTDB S*. *somaliensis* (GCF_000258595)	*GTDB S*. *somaliensis* (GCF_012396115)	91.79	90.1	43.3

**Fig 3:**
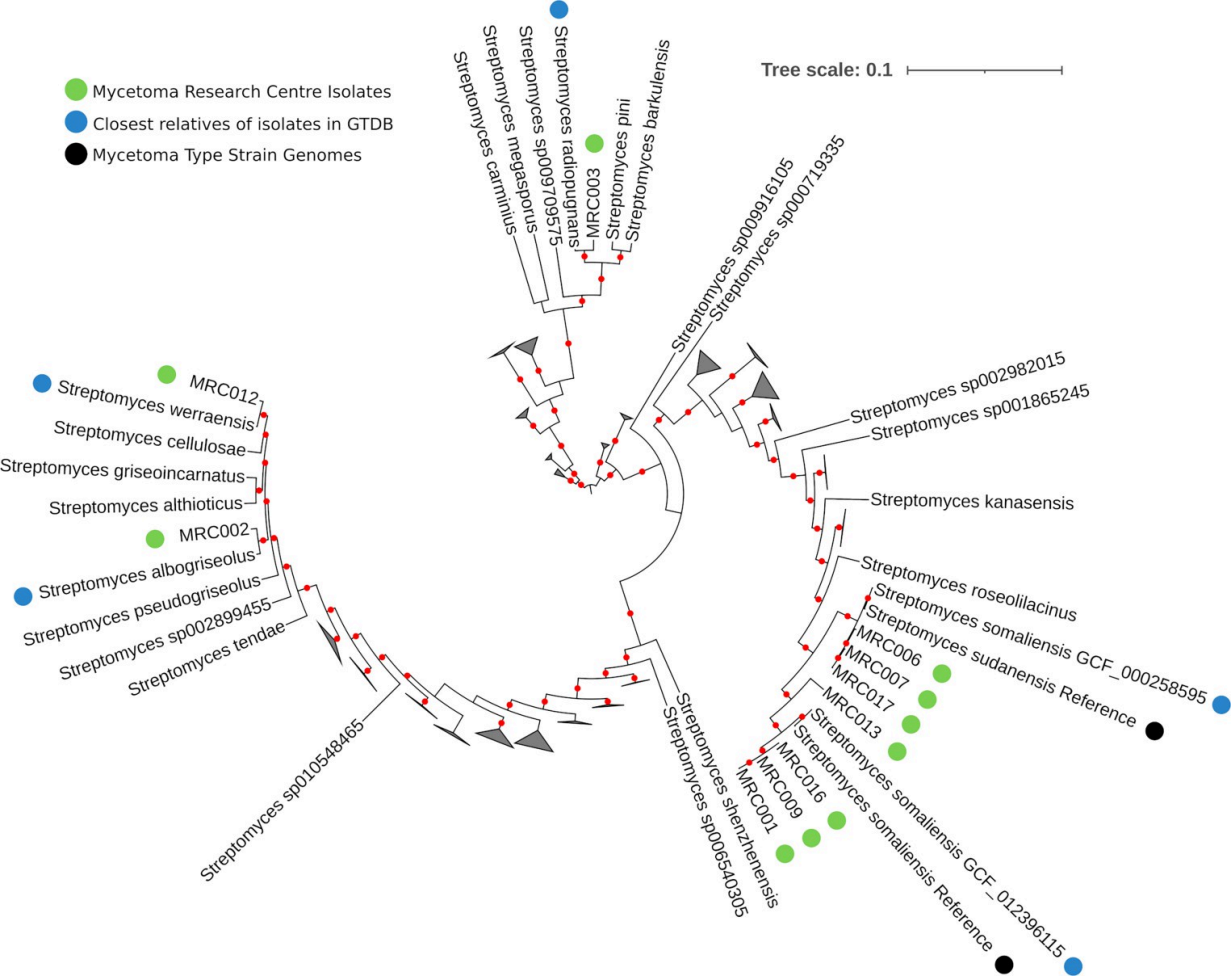
Tree of single copy orthologs belonging to the GTDB bac120 dataset, from the genomes of *Streptomyces* related isolates from the Mycetoma Research Centre (green), their relatives according to ANI in GTDB (with the closest relatives indicated in blue) and genomes from type strains of species typically associated with actinomycetoma (black). A red dot on branches indicates ultrafast bootstrap support >95. Triangles are used to represent collapsed clades. The tree was inferred in iqtree2 using the C20 model. The full figure is available at https://itol.embl.de/shared/1MX60mtB0Ohk3.

**Fig 4:**
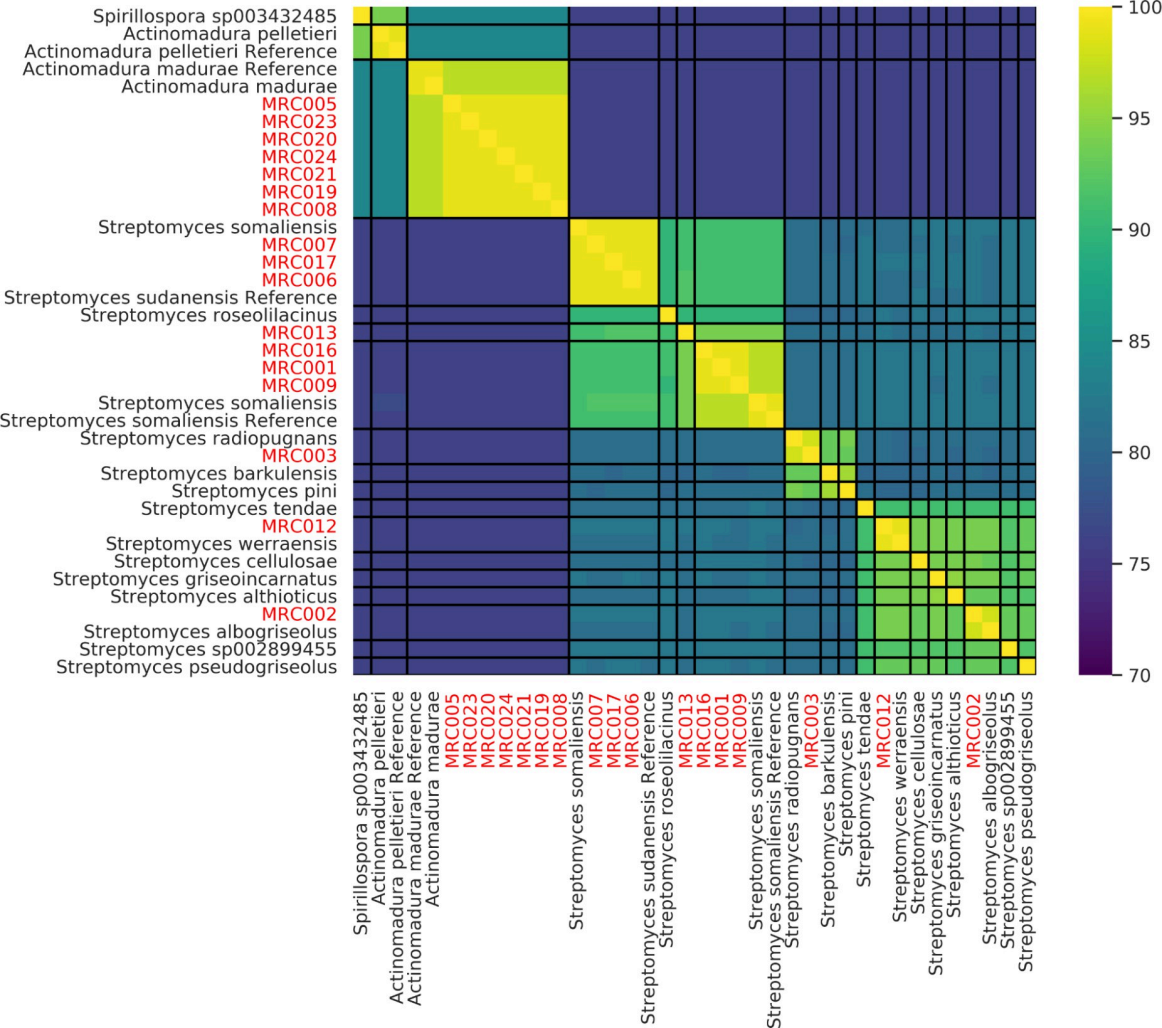
Pairwise comparison of Average Nucleotide Identity between the genomes of Isolates from the Mycetoma Research Centre (red) and their closest relatives in GTDB (all genomes from GTDB with an ANI >90 with any single isolate genome or the genome of type strains). The heatmap is ordered based on hierarchical clustering (Ward, Euclidean distance). Black lines delineate species boundaries based on ANI > 95%. The genomes of the reference species are from type strains.

We used GTDB-tk to provide an initial taxonomic assignment of isolates identified by 16S rRNA analysis as related to *S*. *somaliensis* or *S*. *sudanensis*, and to identify all close relatives of isolates with publicly available genomes deposited in the GTDB (S1 Table). We used these data to construct a multi-marker phylogeny for isolates and their close relatives, and for pairwise comparisons of ANI, AAI and DDH similarities. The data from whole genome comparisons between the isolates and the *S*. *somaliensis* and *S*. *sudanensis* strains were consistent with the results from the 16S rRNA analyses. All three isolates assigned to *S*. *sudanensis* in the 16S rRNA phylogeny were found to be most similar to *S*. *sudanensis* (GCF_000258595.1) by GTDB-tk and formed a strongly supported clade with the recently sequenced *S*. *sudanensis* type strain and the genome GCF_000258595.1 in the multi-marker phylogeny, further supporting the proposal that GCF_000258595.1 [68] is likely to be an *S*. *sudanensis* genome (Fig 3). All ANI, AAI and DDH values between these isolates and the *S*. *sudanensis* genome GCF_000258595.1 were consistent with them belonging to the same species (Table 2 and Figs 4 and S2). Similarly, all four isolates assigned to *S*. *somaliensis* in the 16S rRNA gene sequence analysis formed a strongly supported lineage with *S*. *somaliensis* in the multi-marker tree of the group (Fig 3). In addition, three of the four isolates (the exception MRC013; is discussed below) were also assigned to *S*. *somaliensis* by GTDB-tk; the ANI, AAI and DDH values between these isolates and the type strains of *S*. *somaliensis* supported their assignment to this species (Table 2 and Figs 4 and S2).

**Table 2:** Summarising the pairwise ANI, AAI and estimated DDH values between isolates and their nearest relatives with sequenced genomes. *S*. *somaliensis* and *S*. *sundanensis* type strain genomes are compared to genomes in GTDB. Values that fall below defined thresholds for assigning strains to the same species are highlighted in grey.

Genome 1	Genome 2	ANI (%)	AAI (%)	DDH (GGDH)
MRC001	*S*. *somaliensis* (GCF_012396115)	97.7	97.6	80.5
MRC002	*S*. *albogriseolus* (GCA_014650475)	98.4	98.2	85.1
MRC003	*S*. *radiopugnans* (GCF_900110735)	98.6	98.0	83.5
MRC005	*A*. *madurae* (GCF_900115095)	97.9	97.7	81.6
MRC006	*S*. *sudanensis* (GCF_000258595)	99.0	98.8	89.7
MRC007	*S*. *sudanensis* (GCF_000258595)	99.0	98.9	89.8
MRC008	*A*. *madurae* (GCF_900115095)	97.9	97.6	81.7
MRC009	*S*. *somaliensis* (GCF_012396115)	97.8	97.7	80.4
MRC012	*S*. *werraensis* (GCA_014656175)	99.1	99.0	92.5
MRC013	*S*. *somaliensis* (GCF_012396115)	94.2	93.0	52.9
MRC016	*S*. *somaliensis* (GCF_012396115)	97.6	97.6	80.7
MRC017	*S*. *sudanensis* (GCF_000258595)	99.1	98.9	89.9
MRC019	*A*. *madurae* (GCF_900115095)	97.8	97.5	81.0
MRC020	*A*. *madurae* (GCF_900115095)	97.8	97.5	81.3
MRC021	*A*. *madurae* (GCF_900115095)	97.8	97.5	81.2
MRC023	*A*. *madurae* (GCF_900115095)	97.8	97.5	81.1
MRC024	*A*. *madurae* (GCF_900115095)	97.8	97.6	81.3

### *Streptomyces* strain MRC013, a new actinomycetoma pathogen

Interestingly, strain MRC013 was an outlier within the *S*. *somaliensis* clade in both the 16S rRNA (Fig 1A) and the concatenated protein marker tree (Fig 3). In both trees it formed a strongly supported clade with the type strain of *S*. *somaliensis* and related isolates, but branched at the base of the group as an adjacent lineage. In the 16S rRNA gene tree, this isolate formed a strongly supported clade with a sequence previously isolated from a mycetoma patient (Sengupta, Goodfellow & Hamid NCBI accession: EU544239.1), adjacent to but excluding other isolates and the type strain rRNA. Whole genome comparisons support the annotation of MRC013 as a new species, as do low ANI, AAI and dDDH values of 94.2%, 93.0% and 52.9% with the genome of its closest relative, *S*. *somaliensis* (Table 2 and Figs 4 and S2). Further, pairwise ANI and AAI comparisons of all isolate genomes revealed that MRC013 shares <95% ANI and <95% AAI with the other *Streptomyces* related genomes identified in the GTDB (Figs 4 and S2). These data indicate that MRC013 is a member of a previously unrecognised *Streptomyces* species that is closely related to *S*. *somaliensis* and is a causal agent of human actinomycetoma. Based on its isolation from the region of West Kordofan, the name proposed for this new taxon is *Streptomyces kordofanensis*.

### The first reported isolation of members of three validly published *Streptomyces* species from actinomycetoma patients

The initial taxonomic assignment of three isolates from the genus *Streptomyces*, MRC002, MRC003 and MRC012, did not correspond to previously identified sources of human actinomycetoma. MRC002 16S rRNA was most similar to 16S rRNA from *Streptomyces atrovirens* in the EzBiocloud database, and phylogenetic analysis of 16S rRNA placed it in a lineage with this species and soil isolates from Sudan (Fig 1B), though support for this clade was relatively low (88 ultrafast bootstrap support). In contrast, all whole-genome comparisons including GTDB-tk, the concatenated protein marker tree (Fig 3), ANI, AAI and DDH similarities (Table 2) support the alternative annotation of MRC002 as *Streptomyces albogriseolus*, which was proposed for a neomycin producing strain originally isolated from soil [69].

Both 16S rRNA and all whole-genome and ANI and dDDH data (Figs 1D and 3 and Table 2) support the annotation of MRC003 as *Streptomyces radiopugnans*. The type strain of this species was isolated from a radiation-polluted soil in China and found to be markedly resistant to gamma radiation [70]. Halotolerant strains assigned to this species have been isolated from soil samples from Antarctica [71]. Similarly, corresponding data (Figs 1E and 3 and Table 2) support the assignment of MRC012 to *Streptomyces werraensis* [72], the type strain of which produces nonactin, a polyketide antibiotic. Additional strains assigned to this species have been isolated from soil and animal fecal samples in India [72,73]. It is particularly interesting that isolate MRCO12 forms a well-defined clade (Fig 1E) with strains isolated from soil samples from Sudan and from an organism isolated from lesions of a donkey with withers (EU544241.1;), as mentioned above.

### Seven geographically separated *Actinomadura madurae* isolates with low genetic diversity

**Fig 5:**
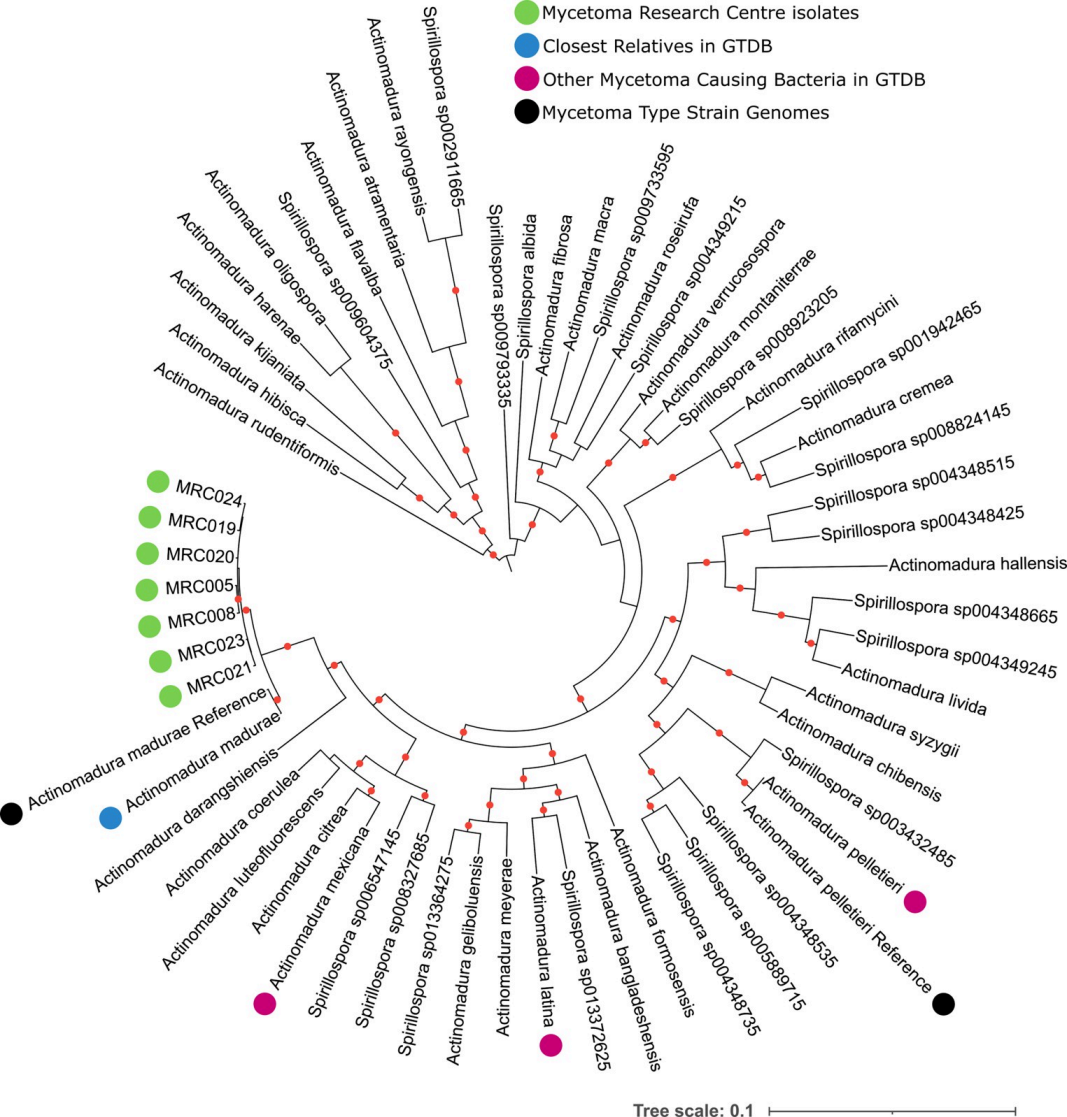
Tree of single copy orthologs that belong to the GTDB bac120 dataset from the genomes of *Actinomadura* related isolates from the Mycetoma Research Centre (green), their relatives according to ANI in GTDB (with the closest relatives indicated in blue) and genomes from type strains of species typically associated with actinomycetoma (black). Additionally, the type strain genome of any organism previously isolated from mycetoma patients and present in the GTDB is highlighted (pink). A red dot on branches indicates ultrafast bootstrap support >95. The tree was inferred in iqtree2 using the C20 model.

Most of the 16S rRNA *Actinomadura* sequences showed their highest similarities to *A*. *mexicana*, whilst in the 16S rRNA tree all samples formed a weakly supported clade that included the type strains of *A*. *madurae* and *A*. *darangshiensis* (Fig 2). Comparative analyses of whole genome sequences are effective in clarifying relationships between closely related species that are difficult to resolve using conventional taxonomic methods [74]. GTDB-tk assigned all isolates to the *A*. *madurae* lineage, a result supported by the concatenated multi-marker phylogeny of the isolates and their close relatives. All of the isolates formed a strongly supported clade with *A*. *madurae*, to the exclusion of *A*. *darangshiensis* (which is a strongly supported adjacent lineage to the *A*. *madurae* / isolate clade) and other characterised *Actinomadura* pathogens such as *A*. *latina*, *A*. *mexicana* and *A*. *pelletieri* (Fig 5). The ANI, AAI and DDH values support the classification of the isolates as *A*. *madurae* (Table 2). Despite the geographic separation of these isolates, MRC019 was from Yemen rather than Sudan, their genomes were highly similar, more so than for any other isolate lineage in the datasets (Figs 4 and S2).

**Fig 6:**
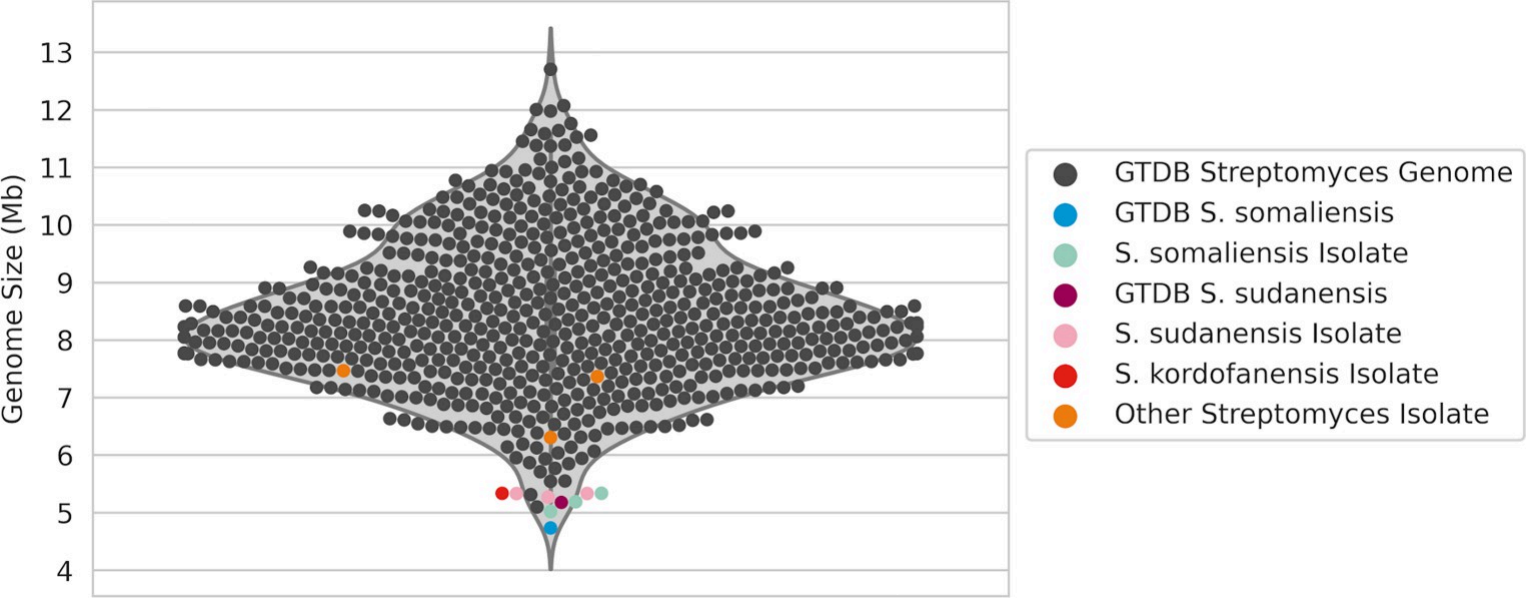
The distribution of genome sizes (in Mb) of all species within the *Streptomyces* genus (as defined by GTDB) for which a high quality genome is available (see materials and methods). The mean genome size for the genus is 8.45Mb. The genomes of all *S*. *sudanensis* and *S*. *somaliensis* related isolates are amongst the smallest genomes in the genus.

Basic statistics on the assembly of all genomes, alongside their final taxonomic assignments, are listed in Table 3. All of these genome sequences are publicly available under NCBI BioProject PRJNA782605. It is interesting that the *S*. *somaliensis* and *S*. *sudanensis* strains have small genomes that range from 5.01 to 5.33 Mbp and 5.27 to 5.37 Mbp, respectively. The corresponding genome size for the putative type strain of *S*. *kordofanensis* is 5.33 Mbp. These are amongst the smallest identified genomes within the *Streptomyces* genus (Fig 6), which averages at 8.45Mb. The closest relatives to *S*. *sudanensis* and *S*. *somaliensis* for which whole genomes are available, as identified by our phylogenetic analyses, are *Streptomyces fradiae* and *Streptomyces roseolilacinus*, both isolated from soil with genome sizes of 6.72Mb and 6.9Mb respectively. The reduction in genome size of members of the *S*. *somaliensis*, *S*. *sudanensis* and *S*. *kordofanensis* clade compared to other *Streptomyces* spp. suggests that these taxa are undergoing an adaption from a saprophytic to a pathogenic lifestyle. It is also interesting that these strains have digital G+C contents that range from 73.93 to 74.18%. In contrast, the *A*. *madurae* strains have much larger genomes and lower G+C contents, as shown in Table 3.

**Table 3:** Assembly statistics for all isolate genomes sequenced in this project. The genomes from these assemblies are available under NCBI Bioproject PRJNA782605.

Isolate Identifier	Species	Genome Size (bp)	Number of contigs	GC content (%)	Long Read Mean Contig Coverage
MRC001	*S*. *somaliensis*	5187137	2	74.18	398
MRC002	*S*. *albogriseolus*	7465071	2	72.51	299
MRC003	*S*. *radiopugnans*	6303566	1	72.98	586
MRC005	*A*. *madurae*	10150872	1	72.14	62
MRC006	*S*. *sudanensis*	5269330	2	74.07	79
MRC007	*S*. *sudanensis*	5331460	2	73.93	79
MRC008	*A*. *madurae*	10232320	1	72.12	86
MRC009	*S*. *somaliensis*	5334785	2	74.15	166
MRC012	*S*. *werraensis*	7363943	2	72.5	230
MRC013	*S*. *kordofanensis*	5333816	1	74.11	204
MRC016	*S*. *somaliensis*	5019138	2	74.01	293
MRC017	*S*. *sudanensis*	5330126	2	73.93	462
MRC019	*A*. *madurae*	10277947	2	72.16	41
MRC020	*A*. *madurae*	10311130	2	72.16	147
MRC021	*A*. *madurae*	10299838	2	72.17	87
MRC023	*A*. *madurae*	10273572	2	72.16	57
MRC024	*A*. *madurae*	10293409	2	72.17	175

### Discrepancies between phenotypic and molecular based classifications

The initial taxonomic classification of 8 of the 15 isolates based on histopathology or cytology was not supported by the taxonomic data (Table 4). Taxonomic classifications based on limited numbers of phenotypic traits are of limited reliability, so discrepancies between these original classifications and those from the genomic analyses are not surprising. These discrepancies are noteworthy due to their potential impact on treatment options. In two cases (MRC002 and MRC005), the initial classification as fungal (eumycetoma) rather than bacterial (actinomycetoma) pathogens led to ineffective lengthy treatment with antifungal agents.

**Table 4:** Initial clinical classification of isolates compared to the molecular taxonomic assignments.

Isolate Metadata				Classification		
Strain number	Duration (years)	Lesion Size (cm)	Locality	Histopathology	Cytology	Molecular
MRC001	1	>10	Khartoum	*S*. *somaliensis*	ND	*S*. *somaliensis*
MRC002	7	> 10	White Nile	*S*. *somaliensis*	*Madurella mycetomatis*	*S*. *albogriseolus*
MRC003	1	NK	North Kordofan	*S*. *somaliensis*	*S*. *somaliensis*	*S*. *radiopugnans*
MRC005	1	5–10	Khartoum	ND	*M*. *mycetomatis*	*A*. *madurae*
MRC006	1	5–10	South Darfour	ND	*A*. *madurae*	*S*. *sudanensis*
MRC007	4	< 5	White Nile	*S*. *somaliensis*	ND	*S*. *sudanensis*
MRC008	1	> 10	South Kordofan	ND	*S*. *somaliensis*	*A*. *madurae*
MRC009	30	> 10	White Nile	ND	*S*. *somaliensis*	*S*. *somaliensis*
MRC012	20	> 10	Kassala	ND	*A*. *madurae*	*S*. *werraensis*
MRC013	3	5–10	West Kordofan	*A*. *madurae*	*A*. *madurae*	*S*. *kordofanensis*
MRC016	1	NK	White Nile	*S*. *somaliensis*	*S*. *somaliensis*	*S*. *somaliensis*
MRC017	1.5	> 10	North Kordofan	*A*. *madurae*	ND	*S*. *sudanensis*
MRC019	8	> 10	Yemen	ND	*A*. *pelletieri*	*A*. *madurae*
MRC020	8	> 10	White Nile	*S*. *somaliensis*	ND	*A*. *madurae*
MRC021	4	> 10	North Kordofan	*A*. *madurae*	ND	*A*. *madurae*
MRC023	1	> 10	North Kordofan	ND	*A*. *madurae*	*A*. *madurae*
MRC024	4	5–10	North Kordofan	ND	*A*. *madurae*	*A*. *madurae*

NK, lesion size not known. ND, not determined.

### In vitro and in silico antibiotic resistence profiling

**Fig 7:**
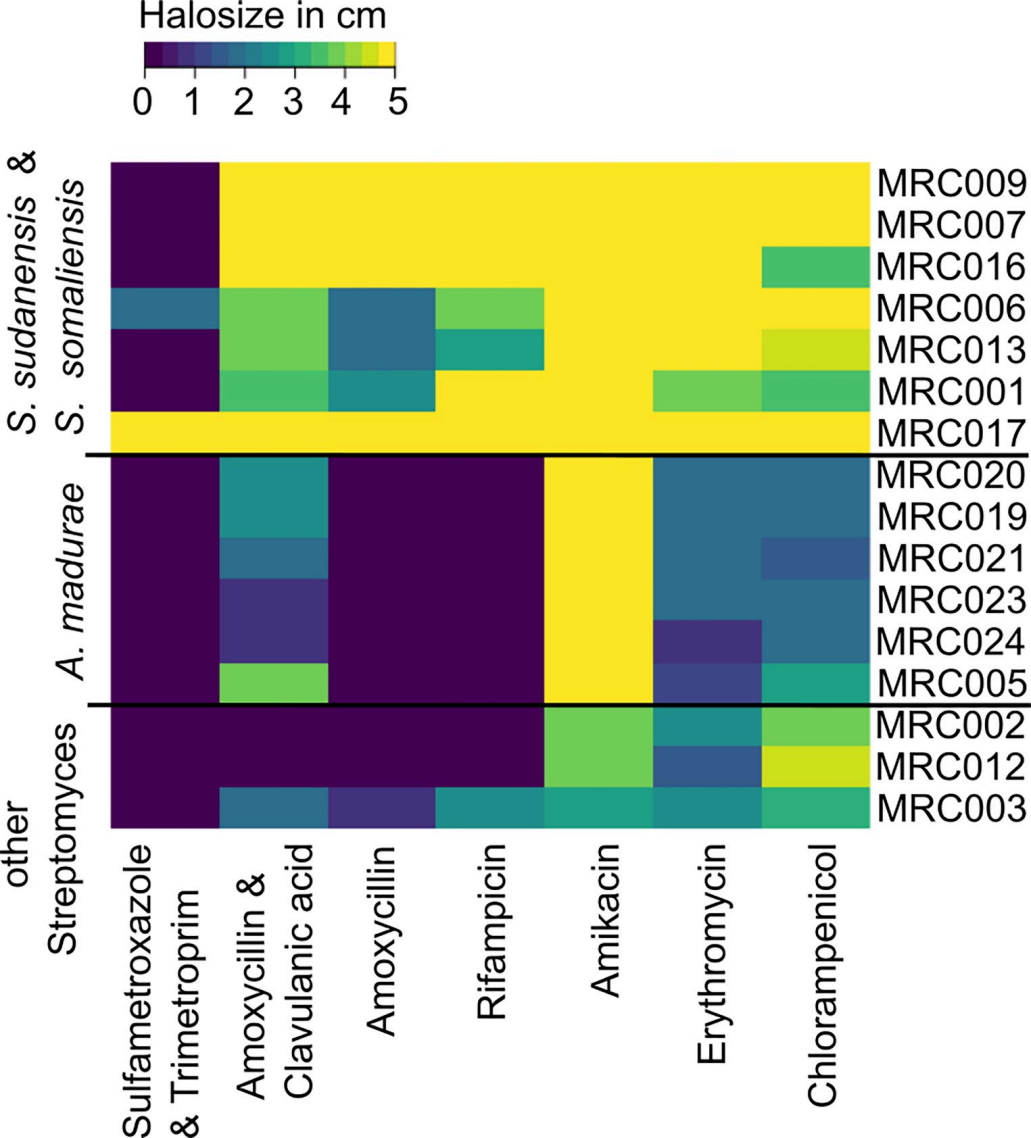
Comparative antibiotic susceptibility profiles of actinomycetoma isolates based on a disk diffusion assay. Halo size was recorded in cm. Yellow low IC. Blue high IC. The isolates were grouped using hierarchical clustering based on their IC profiles using the default parameters of heatmap2 in R.

The first-line treatment of actinomycetoma is a combination of sulfamethoxazole/trimethoprim and amoxicillin/clavulanic acid [36]. All patients from whom samples were isolated in this study received long term treatment amoxicillin/clavulanic acid. To verify the sensitivity of our isolates to these antibiotics and explore potential alternative treatments, we tested each isolate against a range of commonly used antibiotics under laboratory conditions. To our surprise, most isolates showed high ICs to sulfamethoxazole/trimethoprim (Fig 7). In general, the *S*. *somaliensis* and *S*. *sudanenis* strains had a lower IC to the tested antibiotics. The β-lactamase inhibitor clavulanic acid enhanced the sensitivity of most strains to amoxicillin, suggesting that these strains produce a β-lactamase. The *A*. *madurae* isolates had very high ICs to all tested antibiotics, apart from amikacin.

In the light of these findings, we scanned the whole genome sequences for known antibiotic resistance determinants using AMRfinderPLUS (S3 Fig). This indicated the presence of rifampicin resistance genes in *A*. *madurae*, a result in agreement with the observed IC profiles. The *A*. *madurae* strains also had 2 genes annotated as β-lactamases, while the *S*. *somaliensis* and *S*. *sudanensis* strains only had one putatively conferring the higher level of resistance to β-lactam antibiotics observed *in vivo*. These results suggest that whole-genome sequencing can be used to predict antibiotic susceptibility and guide treatment. Alternatively, the distribution of common resistance genes in the genome sequences could be used to generate rapid diagnostic PCR-based tests.

## Discussion

The taxonomic diversity of actinomycetes that can cause actinomycetoma contributes to the difficulty in accurately diagnosing and treating the disease [30,32]. In this study, we compared the currently used phenotypic-based methods for diagnosing mycetoma, such as histopathology, cytology, and micromorphological appearance, with whole-genome sequencing data. While all strains isolated belonged to the phylum *Actinomycetota* [75], formally *Actinobacteria sensu* Goodfellow [76] as predicted by at least one of the classical phenotypic methods, the molecular analysis highlighted several cases in which the causative agent of infection was misdiagnosed. It is also significant that the molecular sequence and genotypic data confirmed the species status of *A*. *latina* [18,19] and *S*. *sudanensis* [23]. Furthermore, the identification of four *Streptomyces* species with no previous association with human mycetoma in this relatively small dataset, including isolate MRC013 which is the first sequenced representative of the presumptive new taxon *S*. *kordofanensis*, expands the range of *Streptomyces* species known to be associated with the disease. Further work with broader sample sizes are needed to establish how important these new species are in the overall worldwide disease burden, and whether more species capable of causing actinomycetoma remain to be discovered, as seems likely [19,77].

The identification of isolates of *S*. *sudanensis* and *S*. *warraenensis* with similarity to strains previously isolated from donkey withers by Sengupta, Goodfellow and Hamid (NCBI accessions EU544231.1 to EU544241.1) suggests that some actinomycetoma causing strains may have the potential to infect multiple host species [48]. Epidemic outbreaks of eumycetoma (*Sporothrix brasiliensis*) have previously been linked with zoonotic transmission from cats to humans [78], and the identification of multi-host actinomycetoma causing strains also raises zoonosis as a possible route for transmission of the bacterial disease.

Actinomycetes are best known for their ability to produce antibiotics and often encompass a multitude of antibiotic resistance genes [79]. To test whether the accurate diagnosis of the causative agent of actinomycetoma infections could positively impact treatment outcomes for the disease, we investigated the IC of isolates to a range of commonly used antibiotics. In general, all strains sequenced showed elevated levels of IC to some antibiotics. It is possible that even higher levels of resistance operate in physiological contexts, for example, within grains. The current first-line treatments for actinomycetoma are long-term administration with sulfamethoxazole/trimethoprim and amoxicillin/clavulanic acid. It was surprising that almost all our isolates had high-level ICs to sulfamethoxazole/trimethoprim under laboratory conditions. In addition, most bacteria had a high IC to amoxicillin. *A*. *madurae* and two of the previously unidentified isolates, MRC002 and MRC0012, which had particularly high ICs, highlighting the potential importance of accurate pathogen identification for treatment choices. Nevertheless, these drugs appear to be effective in a clinical setting, so more work is needed to understand the link between the choice of therapy and the clinical outcome. The activity of amoxicillin could be rescued to a degree by the addition of the β-lactamase inhibitor clavulanic acid, as commonly used in clinical settings. While alternative drugs tested here, such as amikacin, seemed to be more effective, it is important to note that they have considerable toxic side effects [35], hence the benefit may not outweigh the risk for the patient.

In conclusion, the present study shows that the current diagnostic tests used to identify the causative agents of mycetoma have serious limitations. Two of the patients were diagnosed with eumycetoma and given lengthy anti-fungal treatments with common negative side effects and complications, but were thereafter shown to have actinomycetoma. Given the observed differences in antimicrobial IC and misdiagnosis of infections, a wider study of actinomycetoma pathogens from around the world is urgently needed, in combination with the development of point of care rapid molecular diagnostics. Furthermore, antimicrobial IC profile testing should be available at mycetoma clinics to avoid giving patients inappropriate antibiotics, leading to increased morbidity and drug resistance.

The status of *S*. *kordofanensis* as a presumptive new species within the genus *Streptomyces* is mainly based on a comparison of phylogenomic data from MRC013 and of closely related reference strains, notably the type strains of *S*. *somaliensis* and *S*. *sudanensis*.

### Description of Streptomyces kordofanensis sp. nov

#### *Streptomyces* kordofanensis (kor.do.fan.en’ sis. N. L. masc. adj. kordofanensis, belonging to Kordofan, the source of the isolate)

Aerobic, Gram-stain positive actinomycete which forms an extensively branched pale/creamy yellow colour substrate mycelium on Tryptic Soy Agar. Neither aerial hyphae nor diffusible pigments are formed on this medium. Grows from pH6 to pH9, optimally at pH7 from 30 to 37C. Colonies are convex and have filamentous margins. Low IC to amoxicillin (with the effect enhanced by the addition of clavulinic acid), rifampacin, amikacin, erythromycin and chloramphenicol., but had a high IC to sulfamethoxazole and trimetroprim. The digital GC content is 74.11% and the genome length 5.33 Mb, assembled into a single contig.

The type and only strain, MRC013T, was isolated from granulomatous material of mycetoma lesions of a patient in the Sudan. The locus tag of the 16S rRNA gene sequence of the isolate is LUW75_04505 the biosample ID for the genome assembly is SAMN23388489, and the accession is CP094264.

## Supporting information

S1 Fig 16s rRNA phylogeny of isolates from the Mycetoma Research Centre assigned to the genus Streptomyces (green) with related rRNA sequences from the ezBioCloud 16s rRNA database (blue), soil isolates from Sudan (yellow; 48), and isolates collected by Sengupta and Goodfellow from human actinomycetoma (purple) and from donkey withers (lilac). The tree was inferred using iqtree2 under the GTR+F+R5 model and visualised in iTol. Support values correspond to ultrafast bootstraps.
(TIF)

S2 Fig Pairwise comparison of Average Amino Acid Identity (AAI) between the genomes of Isolates from the Mycetoma Research Centre (red) and their closest relatives in GTDB (all genomes from GTDB with an ANI >90 with any single isolate genome or the genome of type strains). The heatmap order matches Fig 4. Black lines delineate species boundaries based on an AAI > 95%. The genomes of the reference species are from type strains.
(TIF)

S3 Fig Antimicrobial resistance prediction using AMRfinderPLUS. The isolates were grouped using hierarchical clustering based on their resistance profile using the default parameters of heatmap2 in R. Color corresponds to the number of genes identified. (white = 0, purple = 1, green = 2, yellow = 3).
(TIF)

S1 Table Output of GTDB-tk, providing a summary of its taxonomic annotation of all isolate and type strain genomes included in this study, as well as listing up to 100 genomes identified as close relatives based on ANI. The standardized bacterial taxonomy established by GTDB, and used by GTDBtk, merges the genus *Spirillospora* with many species typically recognised as belonging to the genus *Actinomadura* in NCBI and LPSN (Approved Lists) [80]. All members of this new merged genus are named *Spirillospora* based on the use of *Spirillospora albus* as the type species for the genus in GTDB. This includes *Actinomadura madurae*, which is renamed to *Spirillospora madurae* in GTDB. All isolates reported as belonging to the *Spirillospora* genus by GTDBtk were positively identified as *Spirillospora madurae* (*Actinomadura madurae*). As such, we elected to continue to use the established genus name for *Actinomadura madurae* to describe the taxonomic assignment of these isolates throughout the manuscript [81], as this name is still recognised as the valid species epithet in LPSN (Approved Lists) and NCBI, and more broadly recognisable in the actinomycetoma research community.
(XLSX)

S2 Table NCBI BioProject and BioSample IDs linked to all genome assemblies presented in this manuscript. (XLSX)

S3 Table SRA metadata linked to the raw sequence data used in the genome assembly of isolates from Sudan. (XLSX)
