# Supplementary figures and images for: Systematic whole-genome sequencing reveals an unexpected diversity among actinomycetoma pathogens and provides insights into their antibacterial susceptibilities
Source: PLoS Negl Trop Dis. 2022 Jul 25;16(7):e0010128. doi: 10.1371/journal.pntd.0010128 (PMC9352199; doi:10.1371/journal.pntd.0010128)

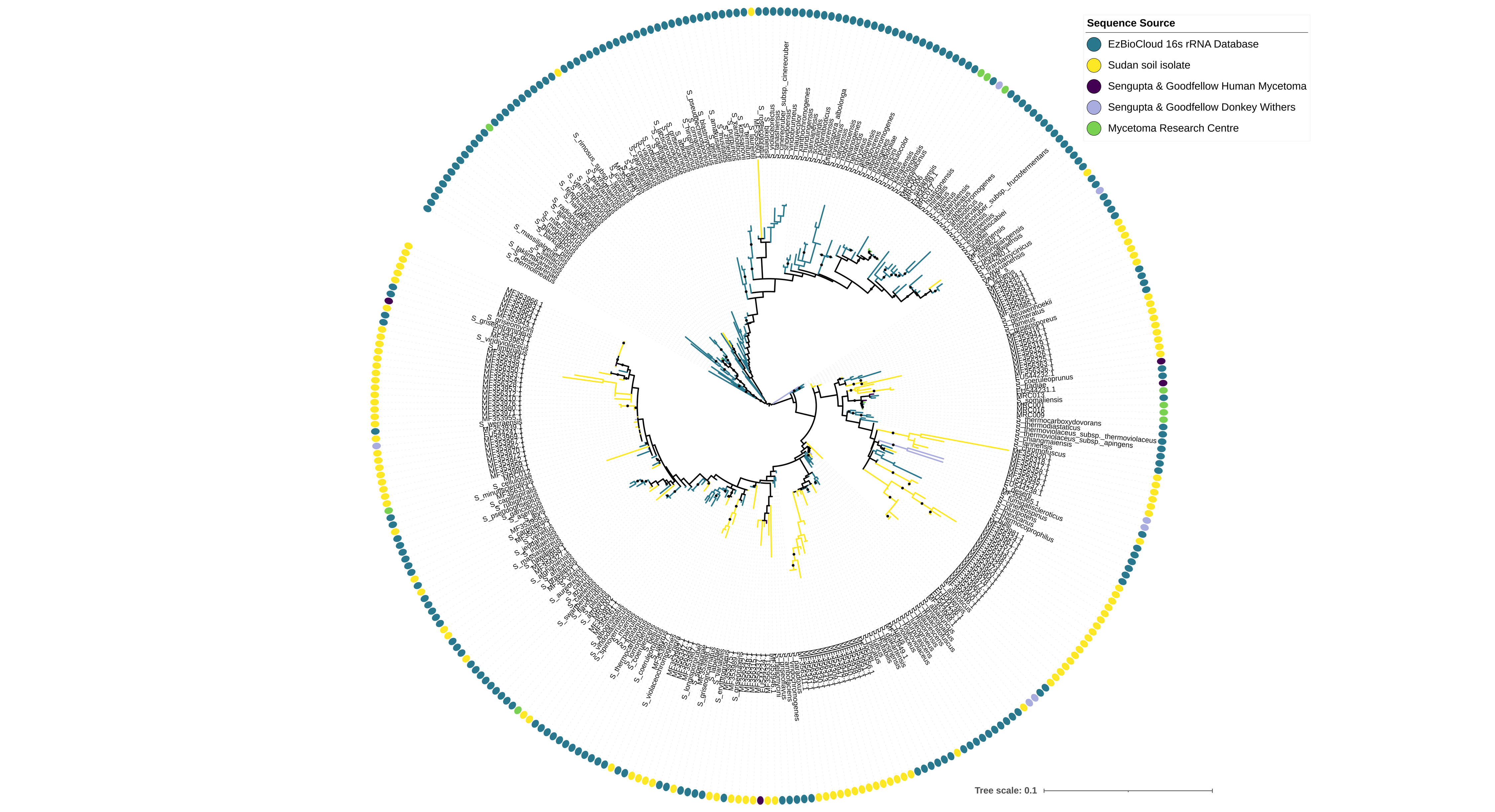

Supplement: S1 Fig — The tree was inferred using iqtree2 under the GTR+F+R5 model and visualised in iTol. Support values correspond to ultrafast bootstraps. (TIF) [file pntd.0010128.s001.tif]

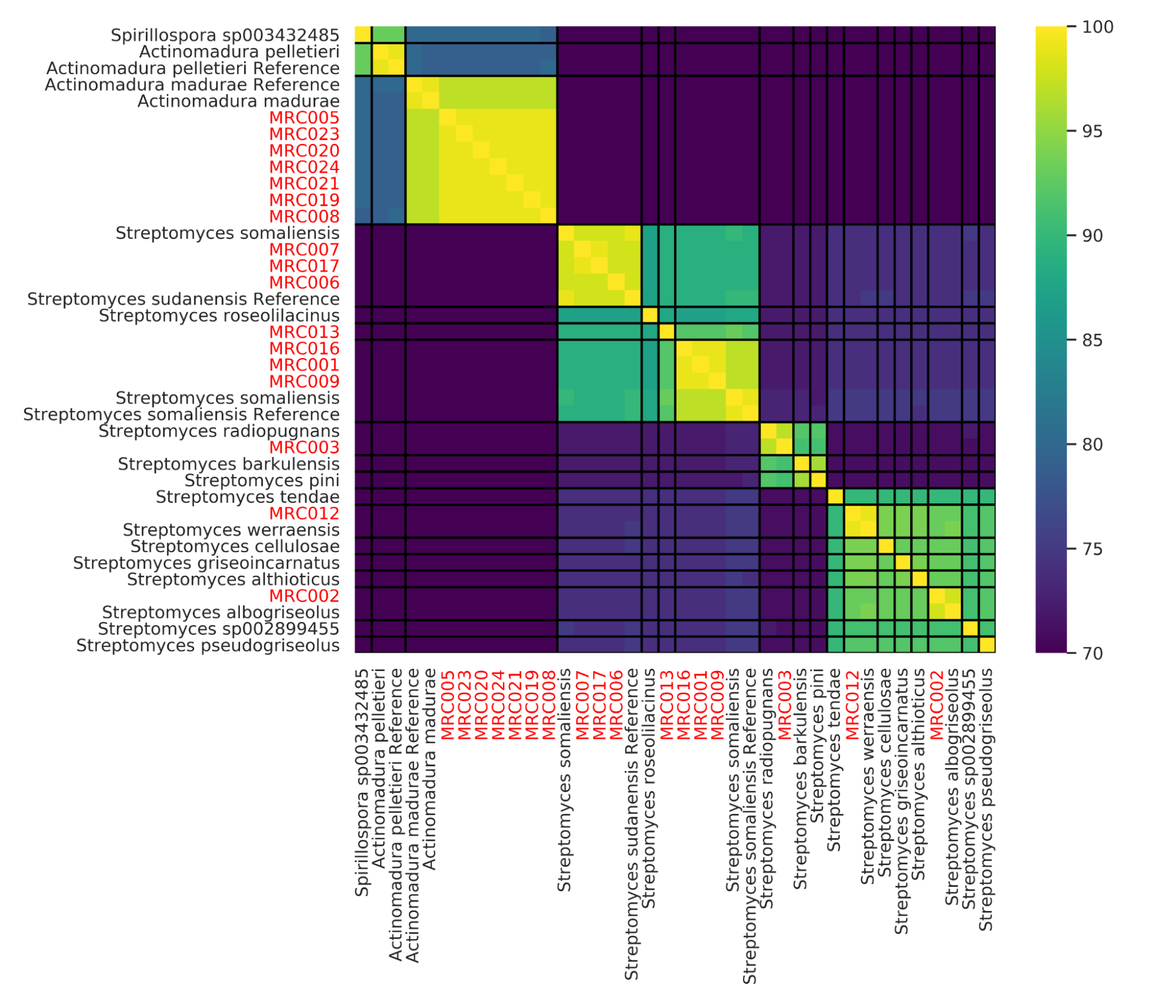

Supplement: S2 Fig — The heatmap order matches Fig 4. Black lines delineate species boundaries based on an AAI > 95%. The genomes of the reference species are from type strains. (TIF) [file pntd.0010128.s002.tif]

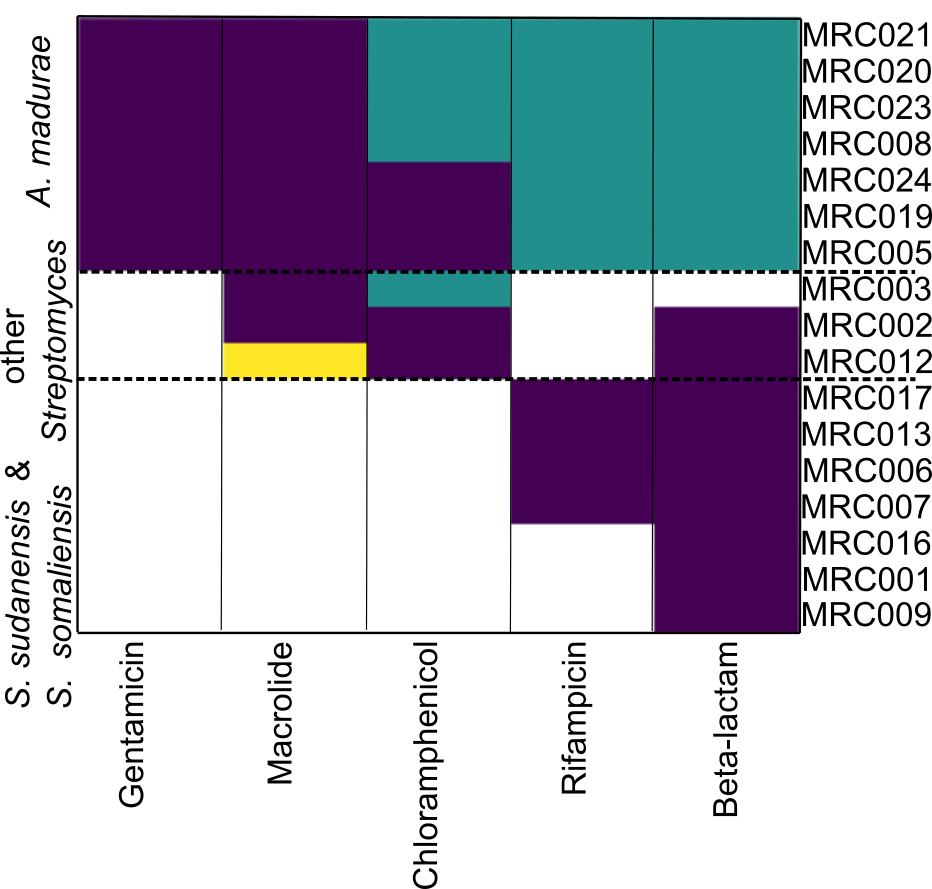

Supplement: S3 Fig — The isolates were grouped using hierarchical clustering based on their resistance profile using the default parameters of heatmap2 in R. Color corresponds to the number of genes identified. (white = 0, purple = 1, green = 2, yellow = 3). (TIF) [file pntd.0010128.s003.tif]
